# Occupational Patterns of Structural Brain Health: Independent Contributions Beyond Education, Gender, Intelligence, and Age

**DOI:** 10.3389/fnhum.2019.00449

**Published:** 2019-12-20

**Authors:** Christian Habeck, Teal S. Eich, Yian Gu, Yaakov Stern

**Affiliations:** Cognitive Neuroscience Division, Department of Neurology, Columbia University, New York, NY, United States

**Keywords:** cortical thickness, occupational data, community cohort, education, age, gender

## Abstract

Occupational activity represents a large percentage of people’s daily activity and thus likely is as impactful for people’s general and cognitive health as other lifestyle components such as leisure activity, sleep, diet, and exercise. Different occupations, however, require different skills, abilities, activities, credentials, work styles, etc., constituting a rich multidimensional formative exposure with likely consequences for brain development over the lifespan. In the current study, we were interested in how different occupations with their different attributes relate to five variables: structural brain health, duration of early-life education, gender, IQ, and age, although the main focus was the relationship to brain health. To this end, we used the Occupation Information Network (O^∗^NET), which provides quantification of occupations along 246 items. Occupational patterns with different loadings for these 246 items were derived from 277 community-dwelling adults, ranging in age from 40 to 80, based upon the five subject measures. We found significant patterns underlying four of our variables of interest, with gender and education predictably showing the most numerous and strongest associations, while brain health and intelligence showed weaker associations, and age did not manifest any associations. For the occupational pattern associated with brain health, we found mainly positive associations on items pertaining to rigorous problem-solving, leadership, responsibility, and information processing. We emphasize that the findings are correlational and cannot establish causation. Future extensions of this work will assess the influence of occupation on future cognitive brain status and cognitive performance.

## Introduction

Occupational attainment and fulfillment has been linked to successful cognitive performance (Garibotto et al., 2008, 2012; Bickel and Kurz, 2009; Foubert-Samier et al., 2012; Pool et al., 2016; Chan et al., 2018; Dodich et al., 2018), psychiatric aging, and general well-being (Dragano et al., 2011; Platts et al., 2013; Wahrendorf et al., 2013) in older adults, independent of socio-economic and educational status.

The relation between occupational attainment/satisfaction and markers of brain-structural health, however, has been probed and observed far less frequently. Some studies have addressed relationships between occupation and brain metabolism (Spreng et al., 2010; Spreng et al., 2011) and white-matter tract integrity (Kaup et al., 2018). In the few studies that have examined cortical thickness and volume, occupation has been shown to be negatively associated with occupational stress (Blix et al., 2013; Savic, 2015; Savic et al., 2018). Negative associations after controlling for clinical disease severity in neurodegenerative disease suggests that occupational attainment is a form of cognitive reserve (Boots et al., 2015).

In the current study, we were interested in the relationship between occupation and structural brain health, with a particular interest in the extent of this relationship beyond possible demographic variables that are collinear confounders of general health status, including age, education, IQ, and gender.

We studied and used occupational attainment as quantified by the extensive characterization in the Occupational Information Network (O^∗^NET^1^), an online resource maintained by the US Department of Labor. Every job, for example Physics Teacher, Postsecondary or Marketing Manager, is assigned a Standard Occupational Classification (SOC) numeric code, 25-1054.00 and 11-2021.00 respectively, and is quantified in terms of a multitude of indicator variables or dimensions. Here, we followed previous research conventions (Peterson and American Psychological Association, 1999; Gadermann et al., 2014) and retained 246 worker-centric variables with data-ratings. These dimensions are drawn from different domains, including ‘work values’ (6 items), ‘interests’ (6 items), ‘knowledge’ (33 items), ‘abilities’ (52 items), ‘work activities’ (41 items), ‘work styles’ (16 items), ‘skills’ (35 items), and ‘work context’ (57 items). To further illustrate this taxonomy, we give a few examples of items in these different categories. ‘Work values,’ ‘interests,’ and ‘knowledge’ are more general and abstract, and thus the labels need more explicit consultation of the online data base. For instance, ‘work values’ concerns items such as ‘achievement’ which specifies an orientation awards results and accomplishments, whereas ‘support’ captures occupations that involve institutionalized support structures (management, HR, etc.). A watch repairer (code 49-9064.00), for instance, would score high on ‘achievement,’ but low on ‘support.’

Domains that are more concrete are ‘work styles,’ ‘work context,’ ‘skills,’ ‘work activities,’ and ‘abilities.’ The labels for the these items are usually self-explanatory, such as ‘Persistence’ (work styles), ‘Contact with others’ (work context),’ ‘Science’ (skills), ‘Interacting with computers’ (work activities), or ‘Memorization’ (abilities). For all results in this paper, every item label will also be supplemented by the appropriate domain label. If the label is not self-explanatory, the exact definition can be looked up in O^∗^NET.

It is noteworthy that the quantification of occupations along the 246 dimensions necessarily induces positive or negative correlations between items. The reasons are twofold: (1) some of the items are intrinsically similar or oppositional. For instance, the complementary work-context item ‘Time spent standing’ can only correlate negatively with ‘Time spent sitting.’ (2) More interestingly, some items are not intrinsically similar or oppositional, but they become so because of the empirical nature of most occupations in our sample. The skill- and work-activities items ‘Critical thinking’ and ‘Handling and moving objects’ are not *a priori* oppositional, and there might be specialized occupations that require both. However, in our sample –and probably the majority of population-based research– they are negatively correlated (*R* = −0.51, *p* < 0.0001). Further, occupational data will most likely be rank-deficient, i.e., the numbers of observations (= participants in sample) might be larger than the number of different occupations. This is the case for our data array, where 277 participants constitute only 152 different occupations.

Apart from inherent correlations between the occupational items, occupational attainment, intelligence, education and brain structural health also usually show mutual associations, and this was no different in our data. Thus, it is difficult isolate a relation between brain health and occupation free from these confounders in cross-sectional associations. At the same time, randomized interventions with occupation are either impossible, or at least only possible in very narrow contexts, and so associational studies have to resort to techniques that try to adjust for the confounders *post hoc*.

In the current study, we investigated the association between a measure of structural brain health and occupational attainment in 246 indicator variables in a community-based cohort of 277 participants, aged 40 to 80. Gender, age, education, and IQ were simultaneously entered with structural brain health as covariates in a general linear model to identify associated items in the occupational data.

## Materials and Methods

### Subject Sample, Acquired Data, and Pre-processing

Participants who lived within a radius of 10 miles of the Columbia University Medical Center were recruited to the study via random market mailing, and were screened for magnetic resonance imaging (MRI) contraindications and hearing or visual impairment that would impede testing. Older adult participants were additionally screened for dementia and mild cognitive impairment prior to participating in the study, and participants who met criteria for either were excluded. Apart from these cognitive exclusion criteria, health-related exclusion criteria included myocardial infarction, congestive heart failure or any other heart disease, brain disorder such as stroke, tumor, infection, epilepsy, multiple sclerosis, degenerative diseases, head injury (loss of consciousness > 5 min), intellectual disability, seizure, Parkinson’s disease, Huntington’s disease, normal pressure hydrocephalus, essential/familial tremor, Down Syndrome, HIV Infection or AIDS diagnosis, learning disability/dyslexia, ADHD or ADD, uncontrolled hypertension, uncontrolled diabetes mellitus, uncontrolled thyroid or other endocrine disease, uncorrectable vision, color blindness, uncorrectable hearing and implant, pregnancy, lactating, any medication targeting central nervous system, cancer within last 5 years, renal insufficiency, untreated neurosyphillis, any alcohol and drug abuse within last 12 month, recent non-skin neoplastic disease or melanoma, active hepatic disease, insulin dependent diabetes, any history of psychosis or ECT, recent (past 5 years) major depressive, bipolar, or anxiety disorder, objective cognitive impairment (dementia rating scale of < 130), and subjective functional impairment (BFAS > 1).

All procedures undertaken for this study were approved by the Columbia Institutional Review Board. Table 1 provides sample information. IQ was assessed with the National Adult Reading Test (Nelson, 1982; Blair and Spreen, 1989).

**TABLE 1:** Number, age, years of education, and IQ of the participant sample.

	**Participant sample**
Age, mean ± STD, range	61.76 ± 9.34, 40–78
Total number, women, men	277, 142 W, 135 M
Self-identified race	67 African American,
	6 Asian,
	189 Caucasian,
	2 Pacific Islander,
	2 Mixed Race,
	11 Other
Education in years, mean ± STD, range	16.31 ± 2.39, 12–22
IQ, mean ± STD, range	118.52 ± 8.99, 93.60–130.88

*IQ was assessed with the National Adult Reading Test.*

#### Occupational Data Acquisition

Comprehensive EXCEL spreadsheets for all 8 domain labels for 969 occupations were downloaded from O^∗^NET in August 2016, then processed and collated following prior established convention (Gadermann et al., 2014). Participants were asked to provide the occupation of the longest duration during their lifetime. A Research Assistant matched the occupation to the O^∗^NET SOC code, and the 246 indicator variables for each code were obtained from the collated spreadsheet.

#### Structural Brain Data Acquisition (T1, DTI, and FLAIR) and Processing

Magnetic resonance imaging images were acquired in a 3.0T Philips Achieva Magnet using a standard quadrature head coil. A T1-weighted scout image was acquired to determine subject position. One hundred sixty-five contiguous 1 mm coronal T1-weighted images of the whole brain were acquired for each subject with an MPRAGE sequence using the following parameters: TR 6.5 ms, TE 3 ms; flip angle 8°, acquisition matrix 256 × 256 and 240 mm field of view. The DTI images were acquired in 55 directions using these parameters: *b* = 800 s/mm^2^, TE = 69 ms, TR = 11032 ms, Flip Angle = 90°, in-plane resolution 112 × 112 voxels, acquisition time 12 min 56 s, slice thickness = 2 mm (no gap), 75 slices. Lastly, a FLAIR scan was acquired with the following parameters: 11,000 ms TR, 2800 ms TE, 256 × 189 voxels in-plane resolution, 23.0 × 17.96 cm field of view (FOV), and 30 slices with slice-thickness/gap of 4/0.5 mm. This sequence was used to quantify the WMHs volumes. A neuroradiologist reviewed each scan individually to exclude any relevant findings. In the case of a clinical positive finding, the subject’s primary care physician was informed.

Each subject’s structural T1 scans were reconstructed using FreeSurfer v5.1^2^. The accuracy of FreeSurfer’s subcortical segmentation and cortical parcelation (Fischl et al., 2002, 2004) has been reported to be comparable to manual labeling. Each subject’s white and gray matter boundaries, as well as gray matter and cerebral spinal fluid boundaries, were visually inspected slice by slice, and manual control points were added in the case of any visible discrepancy. Reconstruction was repeated until we reached satisfactory results within every subject. The subcortical structure borders were plotted by *freeview* visualization tools and compared against the actual brain regions. In case of discrepancy, they were corrected manually. Finally, we obtained cortical thickness for 68 regions of nterest (ROIs), and also read out the main global-thickness value provided by FreeSurfer.

DTI data were processed with TRACULA (Tracts Constrained by Underlying Anatomy) distributed as part of the FreeSurfer v. 5.2 library (Yendiki et al., 2011) which produces 18 major White-Matter tracts. The software performs informed automatic tractography by incorporating anatomical information from a training data set, provided by the software, with the anatomical segmentation of the T1 image of the current data set, thus increasing the accuracy of the WM tract placement for each participant. Standard DTI processing steps using the FMRIB’s Diffusion Toolbox (FMRIB’s Software Library v. 4.1.5) including eddy current correction, tensor estimation, and bedpostx were performed prior to tractography by the TRACULA software (Yendiki et al., 2011). For each participant, the means of fractional anisotropy (FA) for each of the 18 tracts, were entered into subsequent analyses. FA ranges from 0 to 1 with higher number representing more intact WM integrity.

White-Matter-Hyperintensities (WMH) were obtained through segmentation by the Lesion Segmentation Tool algorithm (LST) (Schmidt et al., 2012) as implemented in the LST toolbox version 2.0.15 (June 2017) for Statistical Parametric Mapping (SPM)^3^. The algorithm first segments the T1 images into the three main tissue classes – cerebral brain fluid, gray matter and white matter. Then, this information is combined with the co-registered FLAIR intensities in order to calculate lesion belief maps. By thresholding these maps with a pre-chosen initial threshold, an initial binary lesion map is obtained which is subsequently grown along voxels that appear hyper intense in the FLAIR image. The result is a lesion probability map. Every FLAIR sequence that had a total WMH volume above 1000 mm^3^ was manually inspected to ensure that there were no visible discrepancies. We counted the number of hyper-intense voxels, N, that were classified as hyper intense and transformed as log-WMH = log(N + 1).

### Data Analysis: Multimodal Brain Health Computation

Structural brain health was computed as the multimodal average of global cortical thickness (the total value provided by FreeSurfer, *not* the average of the 68 ROIs), mean tract integrity and the sign-reversed log-WMH measure. Since the three constituents are incommensurate, they were first z-scored and then averaged according to

brainhealth=[z(globalthickness)+z(meantractintegrity) -z(log-WMH)]/3.

We note that our operationalization of this measure is just an obvious starting point in terms of simplicity, but other formulations are conceivable too. Other modalities with differential contributions might be added, optimized for considerations of construct validity beyond this study. Supplementary regression models were run where the brain-health variable was substituted by individual cortical thicknesses. Results can be found in Supplementary Table S2.

### Data Analysis: Mass-Univariate Analysis

We first performed mass-univariate analysis by simultaneously entering all covariates (brain health, education, IQ, gender, age, and race) and performing a linear regression according to

occ(i)=[brain-healtheducationIQgenderagerace1]β+ε

⁢i=1⁢…⁢246.

with a False-Discovery Rate (FDR) of Q < 0.05 (Hochberg and Benjamini, 1990). (**1** denotes the intercept term.) Race was coded as a categorical index array with values of 0 or 1, had 2 columns and 277 rows. Column 1 indicated the status of ‘African American’ (*N* = 67), and column 2 combined the labels ‘Mixed Race,’ ‘Asian,’ ‘Pacific Islander,’ and ‘Other’ (*N* = 21), and thus could be labeled as ‘Neither African American nor Caucasian.’

## Results

We first ran our mass-univariate linear regression with the full covariate set including the racial index array. However, we did not identify any associations between occupation items and race at Q < 0.05, and decided to drop the racial index array from our analyses to increase statistical power. To arrival at our final results, we re-ran the regression models with the reduced set of five covariates: (1) brain health, (2) education, (3) gender, (4) NART-IQ, and (5) age.

### Collinearity of Covariates

To convey an impression of the collinearity of the covariates we report all bivariate correlations at an uncorrected *p*-value of *p* < 0.05. Age displays an expected strong negative correlation with total brain health (*R* = −0.54, *p* < 0.0001) and positive associations with education (*R* = 0.12, *p* = 0.04), NART-IQ (*R* = 0.18, *p* = 0.0025). Lastly, as expected, NART-IQ and education are highly correlated at *R* = 0.54, *p* < 0.0001.

### Univariate Analysis With FDR Correction

We found significant associations at *Q* < 0.05 for all covariates except age. We first turn our attention to the main objective of this study: brain health. We list the 10 strongest associations in Table 2, but give a full listing of the occupational profiles for all covariates in Supplementary Table S1.

**TABLE 2:** Abbreviated listing of up to 10 associations for occupational items and brain health at Q < 0.05.

**Brain health – 39 items in total**			
**Item**	**Domain**	***T***	***p***
**Positive associations**			
AnalyticalThinking	WorkStyles	4.0766	6.01E−05
InformationOrdering	Abilities	3.9901	8.50E−05
AchievementEffort	WorkStyles	3.9348	0.00010586
IdentifyingObjectsActionsandEvents	WorkActivities	3.8641	0.00013957
Support	WorkValues	3.7927	0.00018378
SystemsAnalysis	Skills	3.6278	0.0003415
Mathematics	Knowledge	3.5921	0.00038941
CriticalThinking	Skills	3.5737	0.00041658
MonitorProcessesMaterialsor	WorkActivities	3.4841	0.00057573
Surroundings			
ComplexProblemSolving	Skills	3.4362	0.00068262
**Negative associations**			
ForeignLanguage	Knowledge	–2.7505	0.0063503

*There was only a single negative association, but 38 positive associations in total. The *p*-values listed are uncorrected.*

The items associated with brain health (above and beyond the other covariates) contain a mixture of all domain labels apart from ‘Interests.’ Inspection of all positively correlated items shows work activities, styles and context show items that involve processing of information, numerical reasoning and decision making with the help of computers, facing responsibility and having to show leadership with severe consequence of errors. Numerical and critical-thinking skills and abilities were strongly associated with better brain health too, as were work styles that emphasizes persistence, initiative and leadership. The knowledge item ‘Foreign Language’ showed the only negative association.

For the other covariates, education by far showed the most numerous and significant associations with 180 items (see Supplementary Table S1). We display an abbreviated listing in Table 3, giving the first 10 items in both directions of association.

**TABLE 3:** Abbreviated list of items displaying significant correlations with education at Q < 0.05.

**Education – 180 items in total**			
**Item**	**Domain**	***T***	***p***
**Positive associations**			
WrittenComprehension	Abilities	7.5256	7.78E−13
ActiveLearning	Skills	7.2708	3.84E−12
OralExpression	Abilities	7.2525	4.30E−12
WrittenExpression	Abilities	7.1636	7.43E−12
ReadingComprehension	Skills	7.1374	8.73E−12
Writing	Skills	7.0934	1.14E−11
Speaking	Skills	6.9272	3.12E−11
JudgmentandDecisionMaking	Skills	6.6831	1.33E−10
OralComprehension	Abilities	6.618	1.94E−10
DeductiveReasoning	Abilities	6.4681	4.61E−10
**Negative associations**			
SpendTimeKneelingCrouching	WorkContext	–5.9534	8.11E−09
StoopingorCrawling			
StaticStrength	Abilities	–5.7125	2.93E−08
MultilimbCoordination	Abilities	–5.5934	5.44E−08
SpendTimeBendingorTwistingtheBody	WorkContext	–5.5346	7.36E−08
CrampedWorkSpaceAwkwardPositions	WorkContext	–5.4708	1.02E−07
HandlingandMovingObjects	WorkActivities	–5.4265	1.28E−07
ManualDexterity	Abilities	–5.3087	2.30E−07
SpeedofLimbMovement	Abilities	–5.2865	2.57E−07
ExtentFlexibility	Abilities	–5.2267	3.45E−07
GrossBodyCoordination	Abilities	–5.0671	7.49E−07

*The *p*-values listed are uncorrected.*

Table 3 and the full listing in Supplementary Table S1 show that items pertaining to work-context, -activities, skills and abilities associated with manual labor show a negative association with education, while items associated with white-collar knowledge work are associated positively with education.

Gender shows similarly strong effects, probably expressing stereotypical gender roles with occupation choice that –over time- might reduce. Women choose occupations that show more traditionally female attributes with little constraint by work context, whereas men preferentially have occupations that involve technical expertise, sensory-perception demands and manual labor. We give the abbreviated listing in Table 4.

**TABLE 4:** Abbreviated list of items displaying significant correlations with gender at Q < 0.05.

**Gender – 57 items in total**			
**Item**	**Domain**	***T***	***p***
**Positive associations (i.e., associations with being female)**			
Artistic	Interests	3.5895	0.00039319
Independence	WorkStyles	3.5324	0.00048385
SocialOrientation	WorkStyles	3.5204	0.00050533
Innovation	WorkStyles	3.4141	0.00073776
FineArts	Knowledge	3.3917	0.00079826
SociologyandAnthropology	Knowledge	3.3478	0.00092996
CommunicationsandMedia	Knowledge	3.3209	0.0010205
PhilosophyandTheology	Knowledge	3.3119	0.0010524
Clerical	Knowledge	3.3113	0.0010547
Dependability	WorkStyles	3.2894	0.0011368
**Negative associations (i.e., associations with being male)**			
SoundLocalization	Abilities	–4.3912	1.62E−05
SpatialOrientation	Abilities	–4.2588	2.84E−05
InanOpenVehicleorEquipment	WorkContext	–4.2431	3.03E−05
NightVision	Abilities	–4.1894	3.79E−05
SpendTimeClimbingLaddersScaffoldsor	WorkContext	–4.101	5.44E−05
Poles			
Mechanical	Knowledge	–4.0904	5.68E−05
PeripheralVision	Abilities	–4.0362	7.07E−05
OperatingVehiclesMechanizedDevicesor	WorkActivities	–3.9368	0.00010506
Equipment			
GlareSensitivity	Abilities	–3.8362	0.00015547
Realistic	Interests	–3.7558	0.00021151

*Because gender is coded as Female = 2, Male = 1, positive correlations pertain to items associated with female gender, negative correlations pertain to items associated with male gender. The *p*-values listed are uncorrected.*

Lastly, we list the items associated with crystallized intelligence, i.e., NART-IQ, in Table 5 in full. There were only eight items in total.

**TABLE 5:** Full listing of items associated with NART-IQ at Q < 0.05.

**NART-IQ – 8 items in total**			
**Item**	**Domain**	***T***	***p***
**Positive associations**			
Artistic	Interests	5.1803	4.33E-07
FineArts	Knowledge	4.5252	9.04E-06
Innovation	WorkStyles	4.1979	3.66E-05
ThinkingCreatively	WorkActivities	3.6928	0.00026824
Originality	Abilities	3.5274	0.0004928
**Negative associations**			
Telephone	WorkContext	–3.6853	0.00027582
Integrity	WorkStyles	–3.4688	0.00060787

*The *p*-values listed are uncorrected.*

**FIGURE 1:**
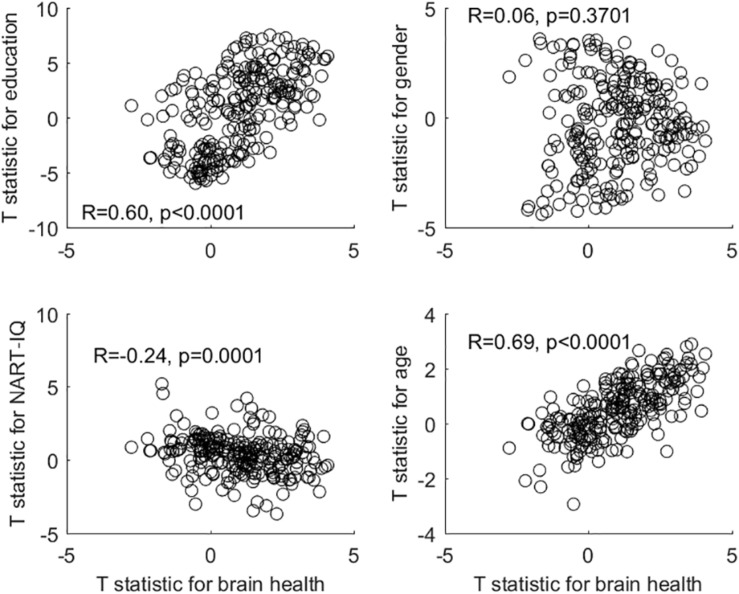
Bivariate plots and correlations between the occupational profile (= T-statistic) of brain health and the occupational profiles of all other covariates.

After deriving the occupational profiles of all covariates, we decided to inspect the similarity between the brain-health occupational profile and all remaining profiles with simple bivariate scatter plots (see Figure 1). This second-order correlation can at least visualize the similarity of the occupation-covariate relationships in relative terms. Interestingly, the brain-health profile shows the greatest similarity to the profiles of education and age (although no individual occupational item showed an association with age at *Q* < 0.05). This similarity is present although the covariates brain health and education showed no relationship, while brain health and age showed a strong *negative* relationship. In our sample at least, older participants chose occupations that are also associated with better brain health and higher education.

The gender-associated occupational profile showed no relationship to the brain-health profile, while the NART-IQ-related profile showed a weak *negative* relationship. At the level of covariates, brain health was unrelated to either gender or NART-IQ.

## Discussion

The main purpose of this study was to clarify the fine-grained relationship between structural brain health and occupation, adjusted for education, gender, age, and IQ. We emphasize again that the results are correlational, and that no inference regarding causal directionality can be made.

The occupational profiles of education, sex, and NART-IQ were somewhat in line with common-sense expectations which would attribute manual-labor and sensory-perception skills and abilities predominantly to male or lower educated participants, while items associated with social orientation, higher knowledge, fine arts and communication were differentially and independently associated with being female and more educated.

For our main association of interest, we mainly found positive associations between items pertaining to processing of information, numerical reasoning, problem-solving and decision making with the help of computers, facing responsibility and having to show leadership with severe consequence of errors. Further correlations were shown with numerical and critical-thinking skills and abilities, and with work styles that emphasize persistence, responsibility, initiative and leadership. Brain health in our operationalization did not show any confounding correlations with education and gender; further, when we ran supplementary analyses leaving out gender and education, the items that were recovered with significant associations were very similar and – in fact-fewer in number. Thus, our results indicate robust relationships between occupation and brain health that are not fully mediated by education, intelligence, or gender.

Furthermore, higher-order correlation of the occupational profiles (= T-statistic) across all 246 items revealed that the brain-health profile was similar (in the sense of being significantly correlated) to the education profile, despite both covariates sharing no significant relationship. There was a likewise similarity between the brain-health occupational profile and the age-related occupational profile, even though brain health and age are strongly *negatively* associated (and no individual item in the age profile reached statistical significance at *Q* < 0.05).

Several caveats must be mentioned in our study design: (1) important information about parental socio-economics and upbringing were missing, although these factors are certain to influence brain development (Noble et al., 2015) beyond the duration of early-life education. To arrive at a relationship between occupation and brain health, this confounder would have to be taken into account. (2) While education and occupation are *not* contemporaneous with, and predate, the brain-health assessment, it is tempting to speculate about causal relationships. It could be that some occupational demands serve as cognitive training regimens that result in better brain health, while some job aspects (particularly environmental exposures) could be detrimental to brain health. However, even for cross-sectional correlations, long-lasting influences of other factors (such as parenting style and early-life socio-economics) would have to be taken into account. To reduce the possibility of reverse causation, i.e., brain health at an early age leading to particular educational and occupational choices, brain health at a young age ideally should also be considered. (3) We only recorded the occupation with the longest tenure in our participants’ lives, and no more detailed information about occupation sequences were queried.

We close our report with some suggestions for future extensions, sparked by the study limitations: a more complete record of occupational history and parental socio-economics is indispensable for a refinement of the relationship between occupation and brain health. Further, while interventional studies for occupation are hard to conceive, prospective cohort studies could record more complete and dynamic occupational information and establish relationships to future brain structural measures, thus getting closer to true a causal account. As mentioned in the introductory remarks, the large amount of time that work represents in the daily routine for most people suggests that occupational choices and demands would be reflected in the brain, similar to other lifestyle features such as exercise, diet, sleep, and leisure activities. To clarify the role of occupation for better brain maintenance and cognitive reserve will be an exciting endeavor in brain research for the foreseeable future.

Our study also hopes to introduce the O^∗^NET database to a broader audience and convey some of the benefits of the fine-grained quantitative assessment of occupation. We only performed simple univariate analyses, which is a natural starting point. O^∗^NET enables more sophisticated frameworks of course, and gives the opportunity of operationalizing similarity and ‘distance’ between occupations, with multivariate decompositions of occupational profiles that capture dimensions other than education, gender, and intelligence. Occupational data might provide a fertile ground for identifying factors with predictive utility for prognosis and diagnosis of cognitive dysfunction in addition to structural brain markers and age.

## Data Availability Statement

The raw data supporting the conclusions of this article will be made available by the authors, without undue reservation, to any qualified researcher. A MATLAB data archive is available with annotation from the corresponding author on request.

## Ethics Statement

The studies involving human participants were reviewed and approved by the Columbia University IRB. The patients/participants provided their written informed consent to participate in this study.

## Author Contributions

CH conceived of the study, analyzed the data, and wrote the draft. TE performed supplementary data analysis and formatting of the occupational data. YG read the manuscript, performed supplementary data analysis, and gave feedback. YS discussed the study extensively, read the draft, and provided commentary.

## Conflict of Interest

The authors declare that the research was conducted in the absence of any commercial or financial relationships that could be construed as a potential conflict of interest.

The handling Editor declared a past co-authorship with one of the authors YS.

## Footnotes

1 https://www.onetonline.org/

2 http://surfer.nmr.mgh.harvard.edu/

3 www.statistical-modelling.de/lst.html
