# Corrigendum: Occupational Patterns of Structural Brain Health: Independent Contributions Beyond Education, Gender, Intelligence, and Age

**DOI:** 10.3389/fnhum.2020.00012

**Published:** 2020-02-26

**Authors:** Christian Habeck, Teal S. Eich, Yian Gu, Yaakov Stern

**Affiliations:** Cognitive Neuroscience Division, Department of Neurology, Columbia University, New York, NY, United States

**Keywords:** cortical thickness, occupational data, community cohort, education, age, gender

In the original article, there was an error. The title was incorrectly given as

“Occupational Patterns of Structural Brain Health: Independent Contributions Beyond Age, Gender, Intelligence, and Age”.

It should be:

“Occupational Patterns of Structural Brain Health: Independent Contributions Beyond Education, Gender, Intelligence, and Age”.

Furthermore, we have replaced “mental retardation” with “intellectual disability”.

The authors apologize for this error and state that this does not change the scientific conclusions of the article in any way. The original article has been updated.

