# Supplementary material for: Occupational Patterns of Structural Brain Health: Independent Contributions Beyond Education, Gender, Intelligence, and Age
Source: Front Hum Neurosci. 2019 Dec 20;13:449. doi: 10.3389/fnhum.2019.00449 (PMC6933301; doi:10.3389/fnhum.2019.00449)
Supplement: Supplementary file 1 [file Table_1.docx]

**6. Supplementary material**

S1: Supplementary Table showing a comprehensive listing of all significant associations for all covariates at Q<0.05. The p-values listed are uncorrected.

| **Item** | **Domain** | **T** | **p** |
| --- | --- | --- | --- |
|  |  |  |  |
| **Education - 180 items** |  |  |  |
| **Positive** |  |  |  |
| WrittenComprehension | Abilities | 7.5256 | 7.78E-13 |
| ActiveLearning | Skills | 7.2708 | 3.84E-12 |
| OralExpression | Abilities | 7.2525 | 4.30E-12 |
| WrittenExpression | Abilities | 7.1636 | 7.43E-12 |
| ReadingComprehension | Skills | 7.1374 | 8.73E-12 |
| Writing | Skills | 7.0934 | 1.14E-11 |
| Speaking | Skills | 6.9272 | 3.12E-11 |
| JudgmentandDecisionMaking | Skills | 6.6831 | 1.33E-10 |
| OralComprehension | Abilities | 6.618 | 1.94E-10 |
| DeductiveReasoning | Abilities | 6.4681 | 4.61E-10 |
| CriticalThinking | Skills | 6.4554 | 4.96E-10 |
| ActiveListening | Skills | 6.4416 | 5.37E-10 |
| InductiveReasoning | Abilities | 6.4222 | 6.00E-10 |
| Achievement | WorkValues | 6.296 | 1.23E-09 |
| EnglishLanguage | Knowledge | 6.2815 | 1.33E-09 |
| CategoryFlexibility | Abilities | 5.9894 | 6.67E-09 |
| WorkingConditions | WorkValues | 5.9478 | 8.36E-09 |
| InterpretingtheMeaningofInformationforOthers | WorkActivities | 5.9171 | 9.86E-09 |
| LearningStrategies | Skills | 5.8614 | 1.33E-08 |
| GettingInformation | WorkActivities | 5.777 | 2.08E-08 |
| AnalyzingDataorInformation | WorkActivities | 5.7592 | 2.29E-08 |
| ComplexProblemSolving | Skills | 5.7507 | 2.39E-08 |
| UpdatingandUsingRelevantKnowledge | WorkActivities | 5.7417 | 2.51E-08 |
| SpeechClarity | Abilities | 5.639 | 4.29E-08 |
| Recognition | WorkValues | 5.6229 | 4.67E-08 |
| AnalyticalThinking | WorkStyles | 5.6117 | 4.95E-08 |
| SystemsAnalysis | Skills | 5.3987 | 1.47E-07 |
| AchievementEffort | WorkStyles | 5.3467 | 1.90E-07 |
| ProvideConsultationandAdvicetoOthers | WorkActivities | 5.3433 | 1.94E-07 |
| InformationOrdering | Abilities | 5.3214 | 2.16E-07 |
| ElectronicMail | WorkContext | 5.3013 | 2.39E-07 |
| Investigative | Interests | 5.229 | 3.41E-07 |
| SystemsEvaluation | Skills | 5.1924 | 4.08E-07 |
| FluencyofIdeas | Abilities | 5.1521 | 4.97E-07 |
| PublicSpeaking | WorkContext | 5.0217 | 9.30E-07 |
| MakingDecisionsandSolvingProblems | WorkActivities | 4.83 | 2.29E-06 |
| Memorization | Abilities | 4.7145 | 3.88E-06 |
| Integrity | WorkStyles | 4.6667 | 4.82E-06 |
| LawandGovernment | Knowledge | 4.6539 | 5.10E-06 |
| NearVision | Abilities | 4.6496 | 5.20E-06 |
| Originality | Abilities | 4.5833 | 6.99E-06 |
| Instructing | Skills | 4.5797 | 7.11E-06 |
| ProblemSensitivity | Abilities | 4.5226 | 9.14E-06 |
| ThinkingCreatively | WorkActivities | 4.4168 | 1.45E-05 |
| Persuasion | Skills | 4.3807 | 1.69E-05 |
| Science | Skills | 4.3744 | 1.74E-05 |
| Initiative | WorkStyles | 4.3701 | 1.77E-05 |
| DevelopingObjectivesandStrategies | WorkActivities | 4.3633 | 1.82E-05 |
| LettersandMemos | WorkContext | 4.3456 | 1.97E-05 |
| ProcessingInformation | WorkActivities | 4.3078 | 2.31E-05 |
| Persistence | WorkStyles | 4.2476 | 2.97E-05 |
| DocumentingRecordingInformation | WorkActivities | 4.2068 | 3.52E-05 |
| EstablishingandMaintainingInterpersonalRelationships | WorkActivities | 4.1225 | 4.99E-05 |
| Monitoring | Skills | 4.1201 | 5.03E-05 |
| FreedomtoMakeDecisions | WorkContext | 4.0677 | 6.23E-05 |
| SociologyandAnthropology | Knowledge | 4.0583 | 6.47E-05 |
| OrganizingPlanningandPrioritizingWork | WorkActivities | 3.9836 | 8.73E-05 |
| EvaluatingInformationtoDetermineCompliancewithStandards | WorkActivities | 3.9107 | 0.00011641 |
| HistoryandArcheology | Knowledge | 3.8398 | 0.00015332 |
| IdentifyingObjectsActionsandEvents | WorkActivities | 3.793 | 0.00018358 |
| TrainingandTeachingOthers | WorkActivities | 3.7852 | 0.00018914 |
| Negotiation | Skills | 3.6784 | 0.00028304 |
| SocialPerceptiveness | Skills | 3.6773 | 0.00028417 |
| EducationandTraining | Knowledge | 3.6483 | 0.00031659 |
| TimeManagement | Skills | 3.6118 | 0.00036227 |
| Independence_2 | WorkValues | 3.5695 | 0.00042292 |
| IndoorsEnvironmentallyControlled | WorkContext | 3.4862 | 0.00057125 |
| PhilosophyandTheology | Knowledge | 3.4325 | 0.00069152 |
| InteractingWithComputers | WorkActivities | 3.4316 | 0.00069378 |
| ResolvingConflictsandNegotiatingwithOthers | WorkActivities | 3.4255 | 0.00070878 |
| SpeedofClosure | Abilities | 3.4166 | 0.00073138 |
| SpendTimeSitting | WorkContext | 3.3823 | 0.00082479 |
| JudgingtheQualitiesofThingsServicesorPeople | WorkActivities | 3.3044 | 0.00108 |
| SpeechRecognition | Abilities | 3.1814 | 0.0016364 |
| SchedulingWorkandActivities | WorkActivities | 3.1586 | 0.0017644 |
| Telephone | WorkContext | 3.1531 | 0.0017969 |
| TherapyandCounseling | Knowledge | 3.1121 | 0.0020564 |
| ComputersandElectronics | Knowledge | 3.0786 | 0.0022929 |
| CoachingandDevelopingOthers | WorkActivities | 3.0537 | 0.0024852 |
| StructuredversusUnstructuredWork | WorkContext | 3.0374 | 0.0026191 |
| PersonnelandHumanResources | Knowledge | 2.9805 | 0.0031393 |
| MathematicalReasoning | Abilities | 2.9326 | 0.003649 |
| OperationsAnalysis | Skills | 2.9215 | 0.0037765 |
| DurationofTypicalWorkWeek | WorkContext | 2.8925 | 0.004132 |
| CommunicatingwithPersonsOutsideOrganization | WorkActivities | 2.8168 | 0.0052077 |
| FacetoFaceDiscussions | WorkContext | 2.7985 | 0.0055028 |
| FlexibilityofClosure | Abilities | 2.6976 | 0.0074223 |
| EconomicsandAccounting | Knowledge | 2.6802 | 0.0078095 |
| Social | Interests | 2.6564 | 0.0083661 |
| Psychology | Knowledge | 2.609 | 0.0095852 |
| Geography | Knowledge | 2.5286 | 0.012019 |
| Leadership | WorkStyles | 2.4617 | 0.014453 |
| CommunicationsandMedia | Knowledge | 2.4377 | 0.015422 |
| AdministrationandManagement | Knowledge | 2.3945 | 0.017322 |
| PerformingAdministrativeActivities | WorkActivities | 2.3631 | 0.018828 |
| AdaptabilityFlexibility | WorkStyles | 2.3536 | 0.019308 |
| SellingorInfluencingOthers | WorkActivities | 2.3526 | 0.019357 |
| ManagementofPersonnelResources | Skills | 2.322 | 0.020972 |
| AttentiontoDetail | WorkStyles | 2.3045 | 0.021949 |
| CoordinateorLeadOthers | WorkContext | 2.3037 | 0.021995 |
| CoordinatingtheWorkandActivitiesofOthers | WorkActivities | 2.2704 | 0.023968 |
| Mathematics_1 | Skills | 2.1955 | 0.028974 |
| ConsequenceofError | WorkContext | 2.1837 | 0.029843 |
| ServiceOrientation | Skills | 2.15 | 0.032444 |
| MonitorProcessesMaterialsorSurroundings | WorkActivities | 2.1348 | 0.033678 |
| **Negative** |  |  |  |
| SpendTimeKneelingCrouchingStoopingorCrawling | WorkContext | -5.9534 | 8.11E-09 |
| StaticStrength | Abilities | -5.7125 | 2.93E-08 |
| MultilimbCoordination | Abilities | -5.5934 | 5.44E-08 |
| SpendTimeBendingorTwistingtheBody | WorkContext | -5.5346 | 7.36E-08 |
| CrampedWorkSpaceAwkwardPositions | WorkContext | -5.4708 | 1.02E-07 |
| HandlingandMovingObjects | WorkActivities | -5.4265 | 1.28E-07 |
| ManualDexterity | Abilities | -5.3087 | 2.30E-07 |
| SpeedofLimbMovement | Abilities | -5.2865 | 2.57E-07 |
| ExtentFlexibility | Abilities | -5.2267 | 3.45E-07 |
| GrossBodyCoordination | Abilities | -5.0671 | 7.49E-07 |
| GrossBodyEquilibrium | Abilities | -5.0389 | 8.57E-07 |
| ArmHandSteadiness | Abilities | -5.0059 | 1.00E-06 |
| OperatingVehiclesMechanizedDevicesorEquipment | WorkActivities | -4.9312 | 1.43E-06 |
| RateControl | Abilities | -4.9229 | 1.48E-06 |
| PerformingGeneralPhysicalActivities | WorkActivities | -4.8677 | 1.92E-06 |
| Stamina | Abilities | -4.8364 | 2.22E-06 |
| SpendTimeKeepingorRegainingBalance | WorkContext | -4.8333 | 2.25E-06 |
| ControlPrecision | Abilities | -4.8166 | 2.43E-06 |
| DynamicStrength | Abilities | -4.7505 | 3.29E-06 |
| PaceDeterminedbySpeedofEquipment | WorkContext | -4.6182 | 5.99E-06 |
| RepairingandMaintainingMechanicalEquipment | WorkActivities | -4.5786 | 7.14E-06 |
| ResponseOrientation | Abilities | -4.5482 | 8.17E-06 |
| ExposedtoContaminants | WorkContext | -4.4913 | 1.05E-05 |
| InanOpenVehicleorEquipment | WorkContext | -4.4399 | 1.31E-05 |
| ReactionTime | Abilities | -4.3435 | 1.98E-05 |
| SpendTimeWalkingandRunning | WorkContext | -4.2952 | 2.43E-05 |
| VeryHotorColdTemperatures | WorkContext | -4.1027 | 5.41E-05 |
| SpendTimeStanding | WorkContext | -4.101 | 5.44E-05 |
| ControllingMachinesandProcesses | WorkActivities | -4.0706 | 6.16E-05 |
| Realistic | Interests | -4.0085 | 7.90E-05 |
| GlareSensitivity | Abilities | -3.9952 | 8.33E-05 |
| ExposedtoHazardousEquipment | WorkContext | -3.976 | 9.00E-05 |
| ExposedtoMinorBurnsCutsBitesorStings | WorkContext | -3.9757 | 9.01E-05 |
| OperationandControl | Skills | -3.9559 | 9.74E-05 |
| TrunkStrength | Abilities | -3.9555 | 9.76E-05 |
| SpendTimeUsingYourHandstoHandleControlorFeelObjectsToolsorContro | WorkContext | -3.8892 | 0.00012658 |
| ExtremelyBrightorInadequateLighting | WorkContext | -3.8801 | 0.00013117 |
| ExposedtoWholeBodyVibration | WorkContext | -3.8772 | 0.00013265 |
| ExposedtoHighPlaces | WorkContext | -3.874 | 0.00013429 |
| WorkSchedules | WorkContext | -3.7953 | 0.00018198 |
| SoundLocalization | Abilities | -3.7771 | 0.00019502 |
| NightVision | Abilities | -3.6558 | 0.00030793 |
| PeripheralVision | Abilities | -3.6206 | 0.00035072 |
| SoundsNoiseLevelsAreDistractingorUncomfortable | WorkContext | -3.6137 | 0.00035979 |
| SpendTimeClimbingLaddersScaffoldsorPoles | WorkContext | -3.4204 | 0.00072155 |
| OperationMonitoring | Skills | -3.4058 | 0.00075969 |
| Troubleshooting | Skills | -3.3503 | 0.00092181 |
| DepthPerception | Abilities | -3.3261 | 0.0010022 |
| ExposedtoHazardousConditions | WorkContext | -3.2808 | 0.0011706 |
| Mechanical | Knowledge | -3.2793 | 0.0011768 |
| SpatialOrientation | Abilities | -3.2635 | 0.0012415 |
| SpendTimeMakingRepetitiveMotions | WorkContext | -3.2194 | 0.001441 |
| ExplosiveStrength | Abilities | -3.1768 | 0.0016614 |
| WearCommonProtectiveorSafetyEquipmentsuchasSafetyShoesGlassesGlo | WorkContext | -3.1584 | 0.0017657 |
| HearingSensitivity | Abilities | -3.0507 | 0.0025093 |
| PhysicalProximity | WorkContext | -3.0058 | 0.0028971 |
| EquipmentMaintenance | Skills | -2.926 | 0.0037241 |
| FingerDexterity | Abilities | -2.8939 | 0.004114 |
| DynamicFlexibility | Abilities | -2.8172 | 0.0052002 |
| RepairingandMaintainingElectronicEquipment | WorkActivities | -2.8004 | 0.0054713 |
| ProductionandProcessing | Knowledge | -2.79 | 0.0056457 |
| QualityControlAnalysis | Skills | -2.7753 | 0.0058992 |
| IndoorsNotEnvironmentallyControlled | WorkContext | -2.7616 | 0.0061452 |
| ImportanceofRepeatingSameTasks | WorkContext | -2.7545 | 0.0062769 |
| Repairing | Skills | -2.6419 | 0.0087229 |
| OutdoorsExposedtoWeather | WorkContext | -2.6182 | 0.0093377 |
| OutdoorsUnderCover | WorkContext | -2.5413 | 0.011602 |
| AuditoryAttention | Abilities | -2.4513 | 0.014864 |
| VisualColorDiscrimination | Abilities | -2.4436 | 0.01518 |
| WristFingerSpeed | Abilities | -2.4097 | 0.016632 |
| Transportation | Knowledge | -2.3591 | 0.01903 |
| FoodProduction | Knowledge | -2.2816 | 0.023287 |
| DraftingLayingOutandSpecifyingTechnicalDevicesPartsandEquipment | WorkActivities | -2.1484 | 0.032564 |
| InspectingEquipmentStructuresorMaterial | WorkActivities | -2.1419 | 0.033092 |
|  |  |  |  |
|  |  |  |  |
| **Gender - 57 items** |  |  |  |
| **Positive - associated with being a woman** |  |  |  |
| Artistic | Interests | 3.5895 | 0.00039319 |
| Independence | WorkStyles | 3.5324 | 0.00048385 |
| SocialOrientation | WorkStyles | 3.5204 | 0.00050533 |
| Innovation | WorkStyles | 3.4141 | 0.00073776 |
| FineArts | Knowledge | 3.3917 | 0.00079826 |
| SociologyandAnthropology | Knowledge | 3.3478 | 0.00092996 |
| CommunicationsandMedia | Knowledge | 3.3209 | 0.0010205 |
| PhilosophyandTheology | Knowledge | 3.3119 | 0.0010524 |
| Clerical | Knowledge | 3.3113 | 0.0010547 |
| Dependability | WorkStyles | 3.2894 | 0.0011368 |
| Social | Interests | 3.2119 | 0.0014779 |
| Relationships | WorkValues | 3.1143 | 0.0020416 |
| SpeechClarity | Abilities | 3.1128 | 0.0020518 |
| AdaptabilityFlexibility | WorkStyles | 3.0501 | 0.002514 |
| Cooperation | WorkStyles | 2.9019 | 0.0040144 |
| ServiceOrientation | Skills | 2.8794 | 0.0043017 |
| SpeechRecognition | Abilities | 2.8075 | 0.0053552 |
| Memorization | Abilities | 2.8057 | 0.0053853 |
| ConcernforOthers | WorkStyles | 2.751 | 0.0063417 |
| PerformingfororWorkingDirectlywiththePublic | WorkActivities | 2.613 | 0.009477 |
| OrganizingPlanningandPrioritizingWork | WorkActivities | 2.6058 | 0.0096723 |
| Writing | Skills | 2.5369 | 0.011746 |
| **Negative - asociated with being a man** |  |  |  |
| SoundLocalization | Abilities | -4.3912 | 1.62E-05 |
| SpatialOrientation | Abilities | -4.2588 | 2.84E-05 |
| InanOpenVehicleorEquipment | WorkContext | -4.2431 | 3.03E-05 |
| NightVision | Abilities | -4.1894 | 3.79E-05 |
| SpendTimeClimbingLaddersScaffoldsorPoles | WorkContext | -4.101 | 5.44E-05 |
| Mechanical | Knowledge | -4.0904 | 5.68E-05 |
| PeripheralVision | Abilities | -4.0362 | 7.07E-05 |
| OperatingVehiclesMechanizedDevicesorEquipment | WorkActivities | -3.9368 | 0.00010506 |
| GlareSensitivity | Abilities | -3.8362 | 0.00015547 |
| Realistic | Interests | -3.7558 | 0.00021151 |
| InspectingEquipmentStructuresorMaterial | WorkActivities | -3.7294 | 0.00023373 |
| BuildingandConstruction | Knowledge | -3.6899 | 0.00027115 |
| DepthPerception | Abilities | -3.634 | 0.00033381 |
| ReactionTime | Abilities | -3.6324 | 0.00033572 |
| Troubleshooting | Skills | -3.594 | 0.00038673 |
| ExposedtoHazardousEquipment | WorkContext | -3.5502 | 0.00045374 |
| EngineeringandTechnology | Knowledge | -3.5093 | 0.00052597 |
| Installation | Skills | -3.5039 | 0.00053619 |
| ExposedtoHighPlaces | WorkContext | -3.4775 | 0.00058939 |
| RateControl | Abilities | -3.337 | 0.00096526 |
| EstimatingtheQuantifiableCharacteristicsofProductsEventsorInform | WorkActivities | -3.3335 | 0.00097716 |
| ControlPrecision | Abilities | -3.3051 | 0.0010773 |
| OperationandControl | Skills | -3.2087 | 0.0014938 |
| RepairingandMaintainingMechanicalEquipment | WorkActivities | -3.1978 | 0.0015492 |
| Physics | Knowledge | -3.1233 | 0.0019822 |
| OutdoorsExposedtoWeather | WorkContext | -3.1047 | 0.0021063 |
| WearSpecializedProtectiveorSafetyEquipmentsuchasBreathingApparat | WorkContext | -3.0452 | 0.002554 |
| TechnologyDesign | Skills | -3.0194 | 0.0027741 |
| ExposedtoWholeBodyVibration | WorkContext | -2.9212 | 0.0037804 |
| Repairing | Skills | -2.8776 | 0.0043262 |
| ExtremelyBrightorInadequateLighting | WorkContext | -2.8622 | 0.0045359 |
| VeryHotorColdTemperatures | WorkContext | -2.8092 | 0.0053277 |
| Investigative | Interests | -2.7955 | 0.0055527 |
| EvaluatingInformationtoDetermineCompliancewithStandards | WorkActivities | -2.573 | 0.010613 |
| EquipmentMaintenance | Skills | -2.5625 | 0.010934 |
|  |  |  |  |
|  |  |  |  |
| **Brain health - 39 items** |  |  |  |
| **Positive** |  |  |  |
| AnalyticalThinking | WorkStyles | 4.0766 | 6.01E-05 |
| InformationOrdering | Abilities | 3.9901 | 8.50E-05 |
| AchievementEffort | WorkStyles | 3.9348 | 0.00010586 |
| IdentifyingObjectsActionsandEvents | WorkActivities | 3.8641 | 0.00013957 |
| Support | WorkValues | 3.7927 | 0.00018378 |
| SystemsAnalysis | Skills | 3.6278 | 0.0003415 |
| Mathematics | Knowledge | 3.5921 | 0.00038941 |
| CriticalThinking | Skills | 3.5737 | 0.00041658 |
| MonitorProcessesMaterialsorSurroundings | WorkActivities | 3.4841 | 0.00057573 |
| ComplexProblemSolving | Skills | 3.4362 | 0.00068262 |
| AnalyzingDataorInformation | WorkActivities | 3.4225 | 0.00071629 |
| WorkingConditions | WorkValues | 3.4015 | 0.0007712 |
| InteractingWithComputers | WorkActivities | 3.3678 | 0.00086753 |
| EstimatingtheQuantifiableCharacteristicsofProductsEventsorInform | WorkActivities | 3.3526 | 0.00091476 |
| SystemsEvaluation | Skills | 3.3042 | 0.0010805 |
| Persistence | WorkStyles | 3.2621 | 0.0012477 |
| Initiative | WorkStyles | 3.2076 | 0.0014994 |
| Achievement | WorkValues | 3.2025 | 0.0015248 |
| JudgmentandDecisionMaking | Skills | 3.2023 | 0.0015262 |
| Recognition | WorkValues | 3.1607 | 0.0017526 |
| OperationsAnalysis | Skills | 3.107 | 0.0020911 |
| ProblemSensitivity | Abilities | 3.0009 | 0.0029426 |
| MakingDecisionsandSolvingProblems | WorkActivities | 2.9998 | 0.0029533 |
| ConsequenceofError | WorkContext | 2.9904 | 0.0030427 |
| ManagementofFinancialResources | Skills | 2.943 | 0.0035322 |
| NumberFacility | Abilities | 2.9319 | 0.0036565 |
| ResponsibilityforOutcomesandResults | WorkContext | 2.9271 | 0.0037121 |
| DeductiveReasoning | Abilities | 2.9169 | 0.0038308 |
| OrganizingPlanningandPrioritizingWork | WorkActivities | 2.8996 | 0.0040426 |
| Monitoring | Skills | 2.8633 | 0.0045207 |
| GettingInformation | WorkActivities | 2.8295 | 0.0050109 |
| PerceptualSpeed | Abilities | 2.8008 | 0.0054654 |
| MathematicalReasoning | Abilities | 2.78 | 0.0058168 |
| Mathematics_1 | Skills | 2.7354 | 0.006642 |
| AttentiontoDetail | WorkStyles | 2.7328 | 0.0066923 |
| ManagementofMaterialResources | Skills | 2.7117 | 0.0071224 |
| Leadership | WorkStyles | 2.7043 | 0.0072773 |
| **Negative** |  |  |  |
| ForeignLanguage | Knowledge | -2.7505 | 0.0063503 |
|  |  |  |  |
|  |  |  |  |
| **NART - 8 items** |  |  |  |
| **Positive** |  |  |  |
| Artistic | Interests | 5.1803 | 4.33E-07 |
| FineArts | Knowledge | 4.5252 | 9.04E-06 |
| Innovation | WorkStyles | 4.1979 | 3.66E-05 |
| ThinkingCreatively | WorkActivities | 3.6928 | 0.00026824 |
| Originality | Abilities | 3.5274 | 0.0004928 |
| **Negative** |  |  |  |
| Telephone | WorkContext | -3.6853 | 0.00027582 |
| Integrity | WorkStyles | -3.4688 | 0.00060787 |

**Supplementary analysis**: Mass-univariate regression models were run with a substitution of the composite brain health score by individual regional thickness. The regression models were

**occ**(i) = [**thickness (**j**) education IQ gender age 1**] **β** + **ε**

i=1…246, j=1…68

where *i* indexes occupational items and *j* indexes cortical regions.

There are 68 regions and 68 mass-univariate regression models were run. FDR correction was performed for the number of items in reach regression model, but *not*  for the number of regions. Only three regions were located with as many occupational items, their associations with select items are listed in table S2 below.

S2: Association between regional thickness and occupational items, after adjusting for age, gender, IQ, and education. Listed are associations that are significant at Q<0.05 (adjusted for 246 occupational items). The p-values listed are uncorrected.

|  | | | |
| --- | --- | --- | --- |
| **Item** | **Domain** | **T** | **p** |
| Region: lh-isthmuscingulate | | | |
| Social | Interests | -3.9061 | 0.00011849 |
| Region: rh-inferiortemporal | | | |
| ConsequenceofError | WorkContext | 3.9028 | 0.00012003 |
| Region: rh-parahippocampal | | | |
| SocialOrientation | WorkStyles | -3.8107 | 0.00017156 |
